# Supplementary material for: Supervised and unsupervised deep learning-based approaches for studying DNA replication spatiotemporal dynamics
Source: Commun Biol. 2025 Feb 26;8:311. doi: 10.1038/s42003-025-07744-2 (PMC11865476; doi:10.1038/s42003-025-07744-2)
Supplement: Supplementary file 1 — Supplemental Material [file 42003_2025_7744_MOESM1_ESM.pdf]

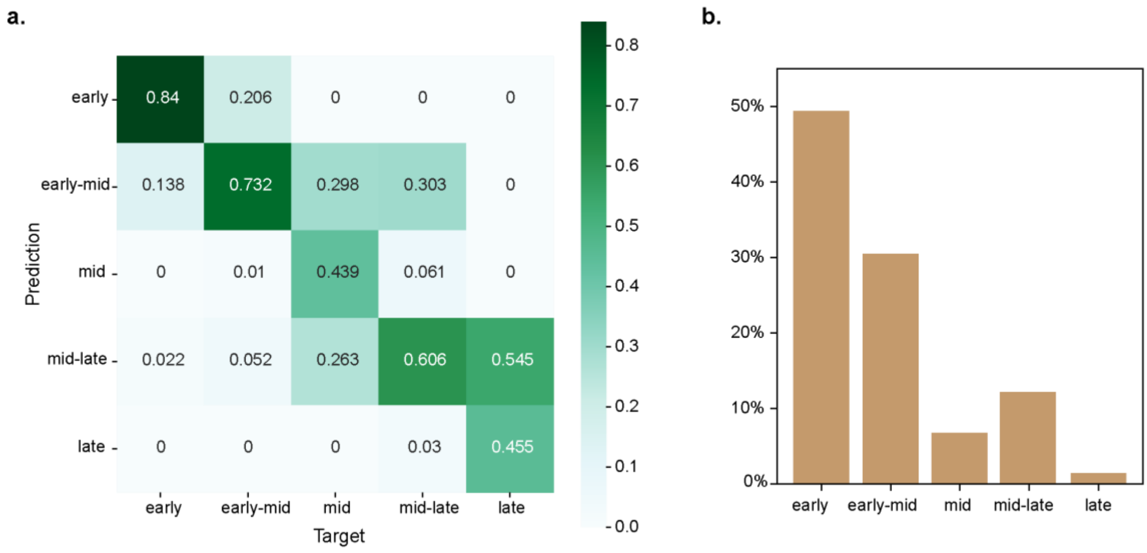

Supplementary Fig. 1. 'S-phase classifier' is robust to small variation in experimental conditions. a. Confusion matrix for evaluation performed on a different test set comprising 423 S-phase nuclei where mESCs were EdU-pulsed for only five minutes. The 'S-phase classifier' achieved comparable accuracies despite not being retrained or fine-tuned on this dataset. The test set distribution according to early, early-mid, mid, mid-late and late stages was as follows: 225, 97, 57, 33 and 11. **b.** Bar plot showing the predicted S-phase distribution, which is consistent with that obtained on the original dataset where mESCs were pulsed with EdU for 30 minutes (Fig. 3a, all the genotypes -OHT and wild type +OHT). Of note, these images were obtained from a separate experiment where cells were not cytospun onto slides.

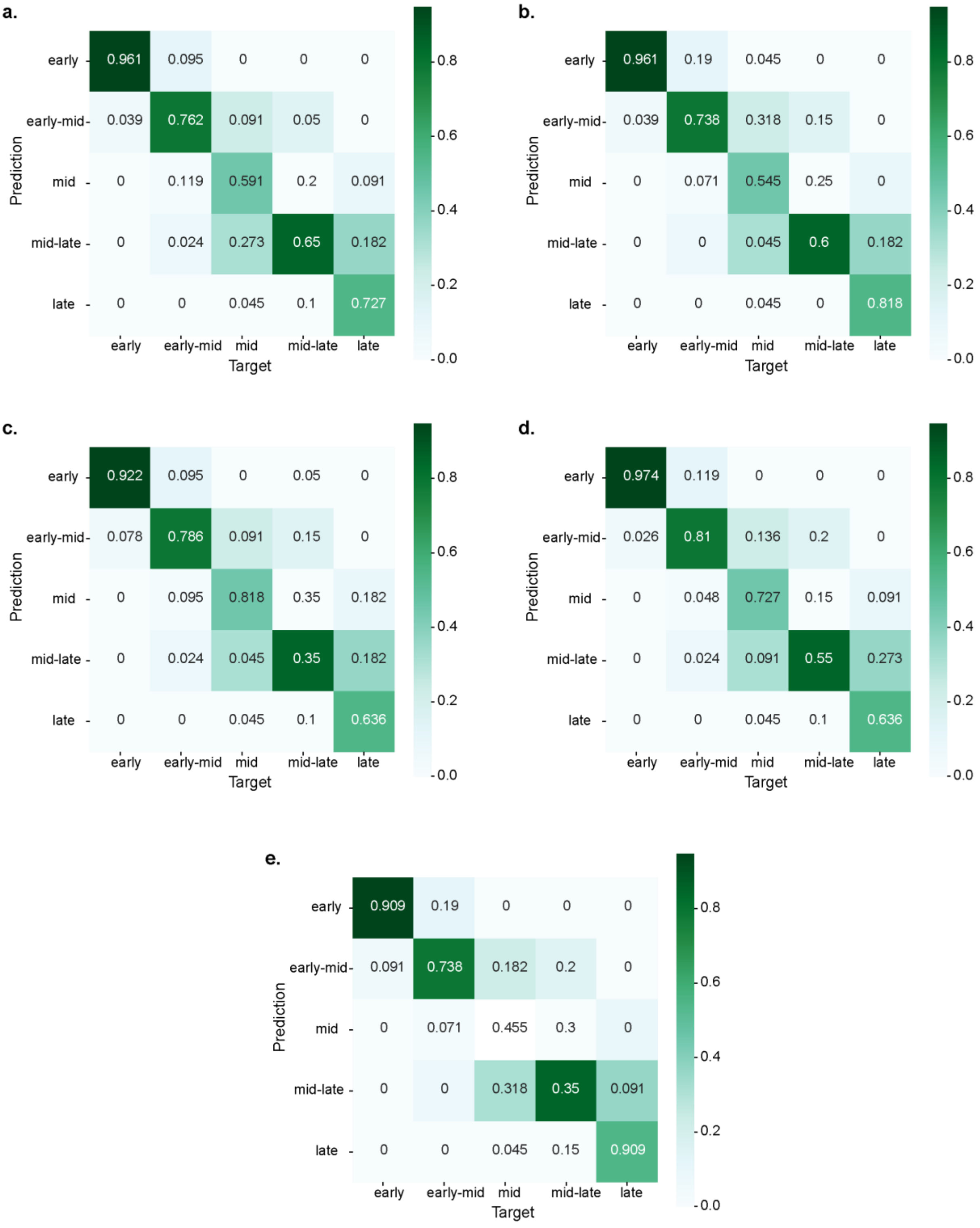

Supplementary Fig. 2. Comparable classification accuracies demonstrated by smaller convolutional neural network architectures. Confusion matrices showing the performance of a pre-trained **a.** ResNet-18 **b.** EfficientNet B0 **c.** MobileNet V2 and **d.** ShuffleNet-V2 convolutional neural network in classifying the different stages of S-phase. The performance of the Boyd *et al.* model[28], which was trained only on the EdU channel, is provided in **e.** Classification performance was evaluated on the same test set as in Fig 1c.

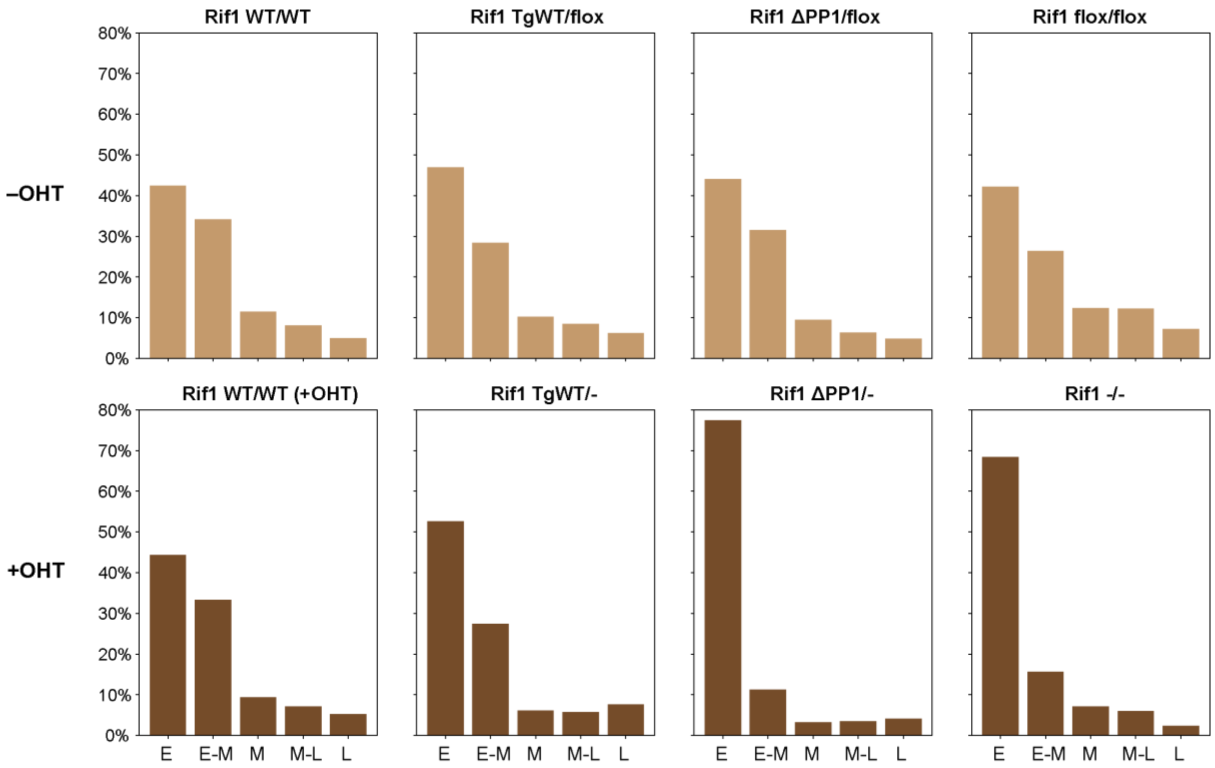

Supplementary Fig. 3. Similar pattern of aberrant DNA replication dynamics identified upon *Rif1* loss of function by a different model trained through supervised learning. Bar plots showing, per genotype, the proportion of nuclei assigned to each of the five categories (early, early-mid, mid, mid-late and late) by the trained Boyd et al. model. The difference in distribution following addition of OHT was statistically interrogated based on Chi-squared tests, yielding the following results: $Rif1^{WT/WT}$ (+OHT): $p = 9.5 \times 10^{-3}$; $Rif1^{TgWT/-}$: $p = 1.2 \times 10^{-9}$; $Rif1^{\Delta PP1/-}$: $p = 1.3 \times 10^{-200}$; $Rif1^{-/-}$: $p = 3.9 \times 10^{-108}$. The top (-OHT) and bottom (+OHT) rows correspond, respectively, to pre- and post-OHT treatment, with all cell lines phenotypically wild-type in the absence of OHT. The different genotypes are described in the 'Cell lines' section of the 'Methods'.

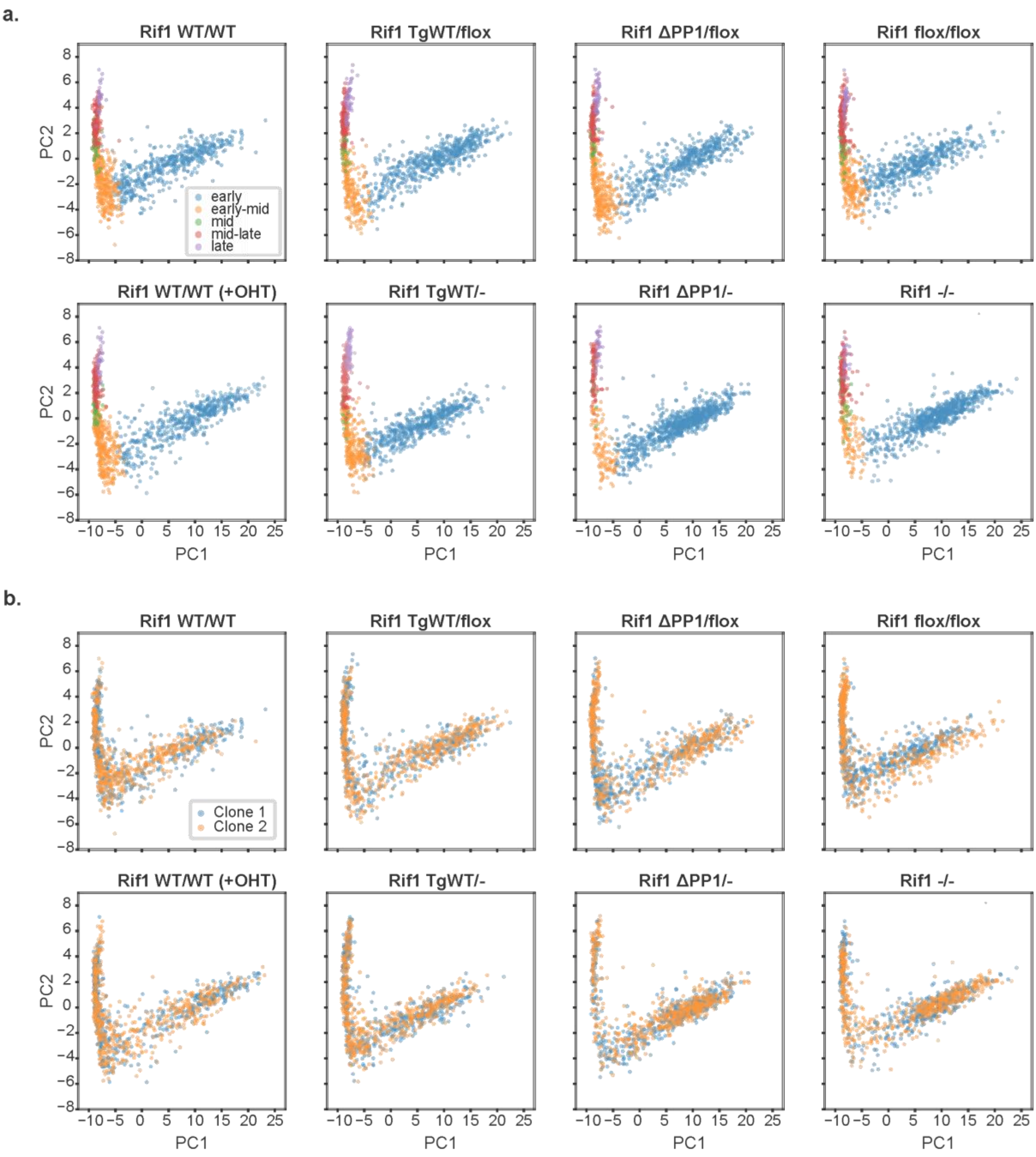

Supplementary Fig. 4. Aberrant DNA replication dynamics in *Rif1* mutant mESCs identified using a supervised approach. Scatter plots of the projected image embeddings, corresponding to the KDE plots in Fig. 3b. An equal number of nuclei per genotype (N = 1,000; 500 for each clone) was randomly sampled to facilitate comparison. The plots are coloured according to **a.** the class predicted by the 'S-phase classifier' and **b.** whether they belong to the first or second clone.

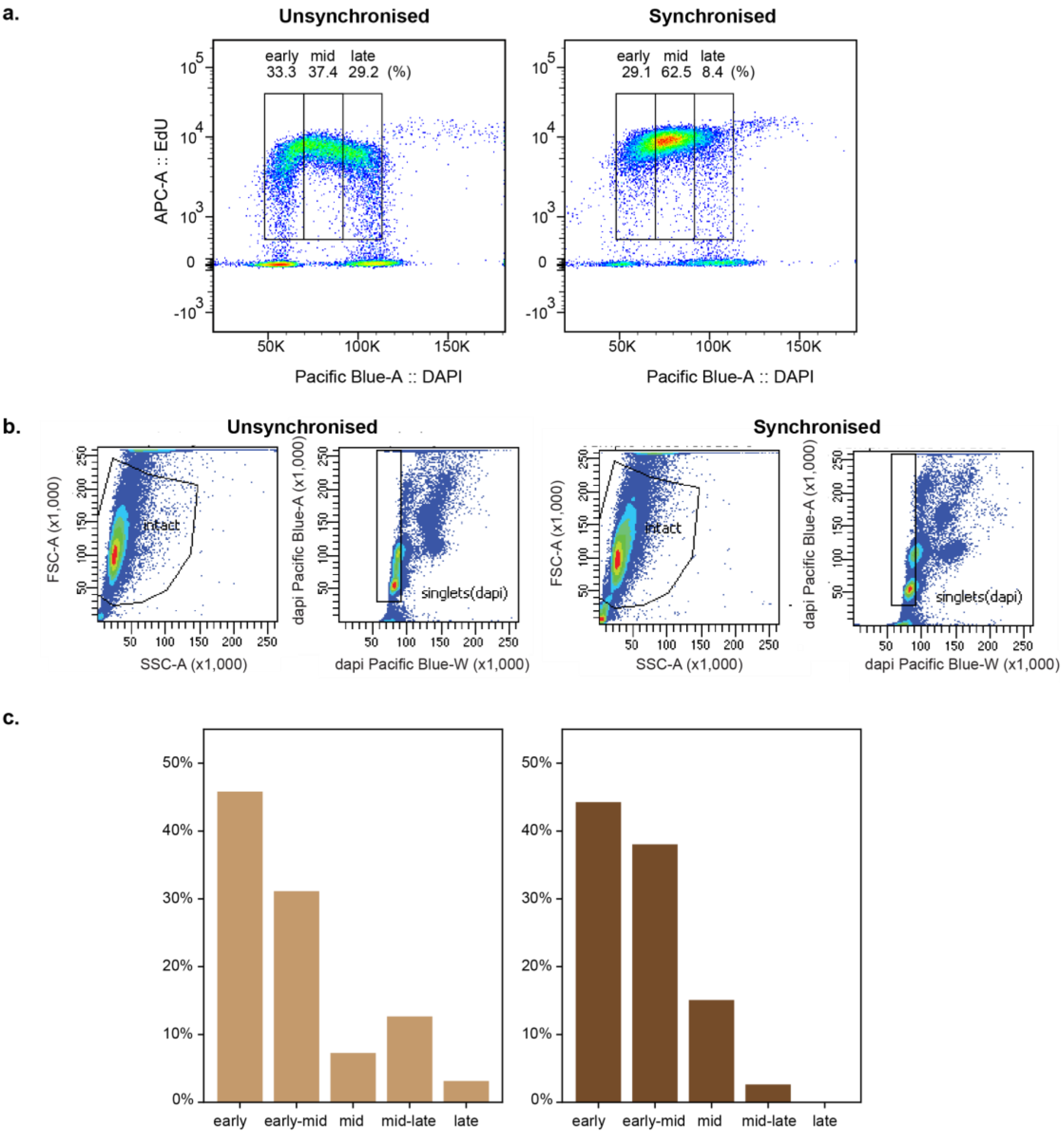

Supplementary Fig. 5. 'S-phase classifier' successfully identifies expected S-phase distribution of synchronised population of mESCs. FACS density plots, indicating the distribution of S-phase nuclei within a representative mESC cycling population from a separate experiment (left), and a synchronized mESC population harvested three hours post-release from thymidine-induced G1/S arrest (right). The allocation of cells into three subpopulations within S-phase was decided according to a split into three equally sized bins, based on DNA and EdU content. Hence, the percentage figures derived do not correlate directly with those obtained through the analysis of replication foci patterns (see **c.**). Nonetheless, these distributions independently confirm the substage shifts in S-phase following synchronization. **b.** FACS gating strategy for the samples shown in a. **c.** The right bar plot shows the predicted distribution when the 'S-phase classifier' was applied to the dataset comprising 305 S-phase nuclei, obtained from the same thymidine-synchronised population. The predicted distribution according to early, early-mid, mid, mid-late and late stages was as follows: 135, 116, 46, 8 and 0. The left bar plot, which corresponds to the leftmost bar plot from Fig 3a., has been included here for comparison. The difference in distribution was statistically significant based on a Chi-squared test ($p = 2.0 \times 10^{-12}$).

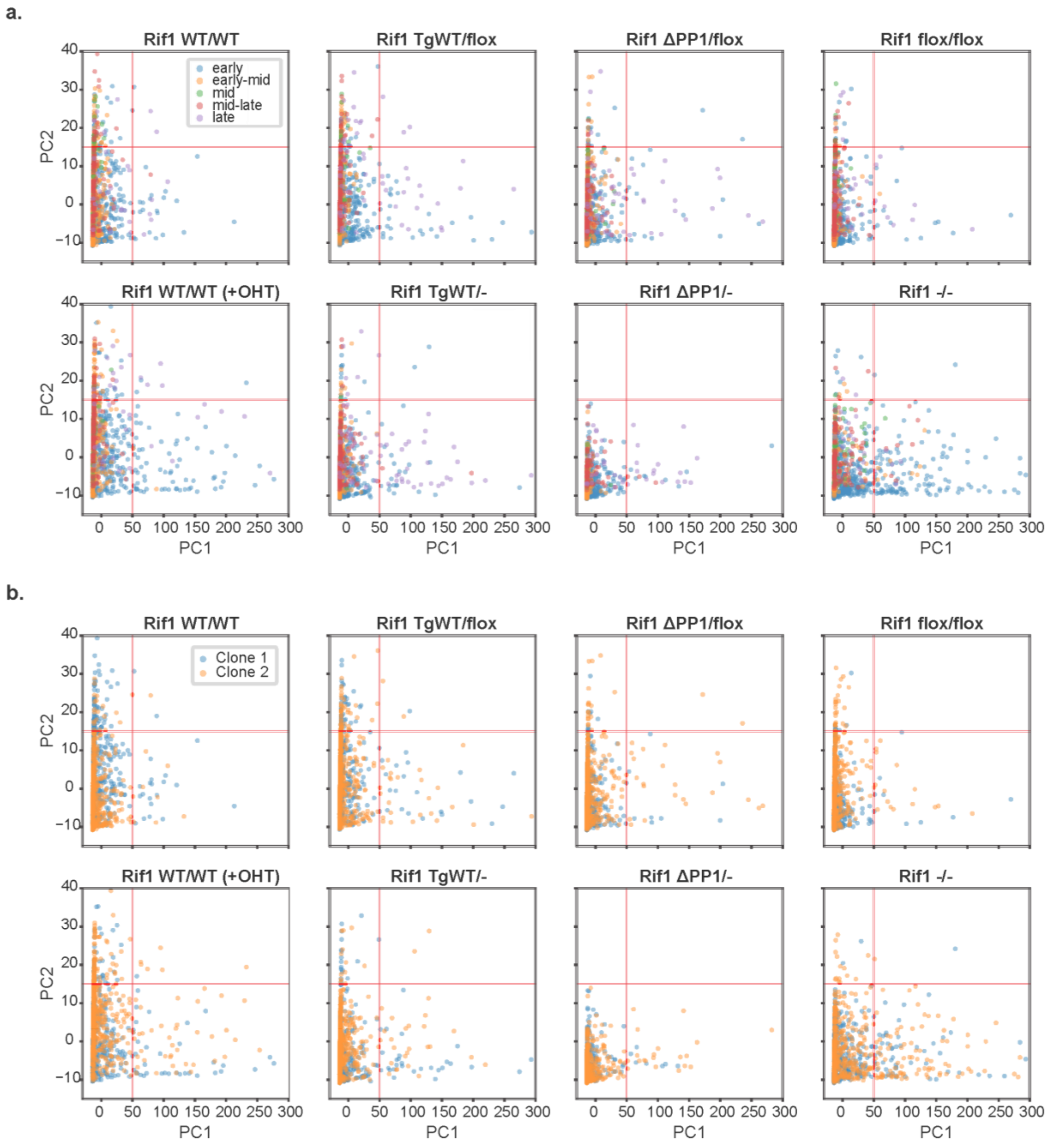

Supplementary Fig. 6. Aberrant DNA replication dynamics in *Rif1* mutant mESCs identified using an unsupervised approach. Scatter plots of the projected image embeddings, corresponding to the KDE plots in Fig. 4b. An equal number of nuclei per genotype (N = 1,000; 500 for each clone) was randomly sampled to facilitate comparison. The plots are coloured according to **a.** the class predicted by the 'S-phase classifier' and **b.** whether they belong to the first or second clone.

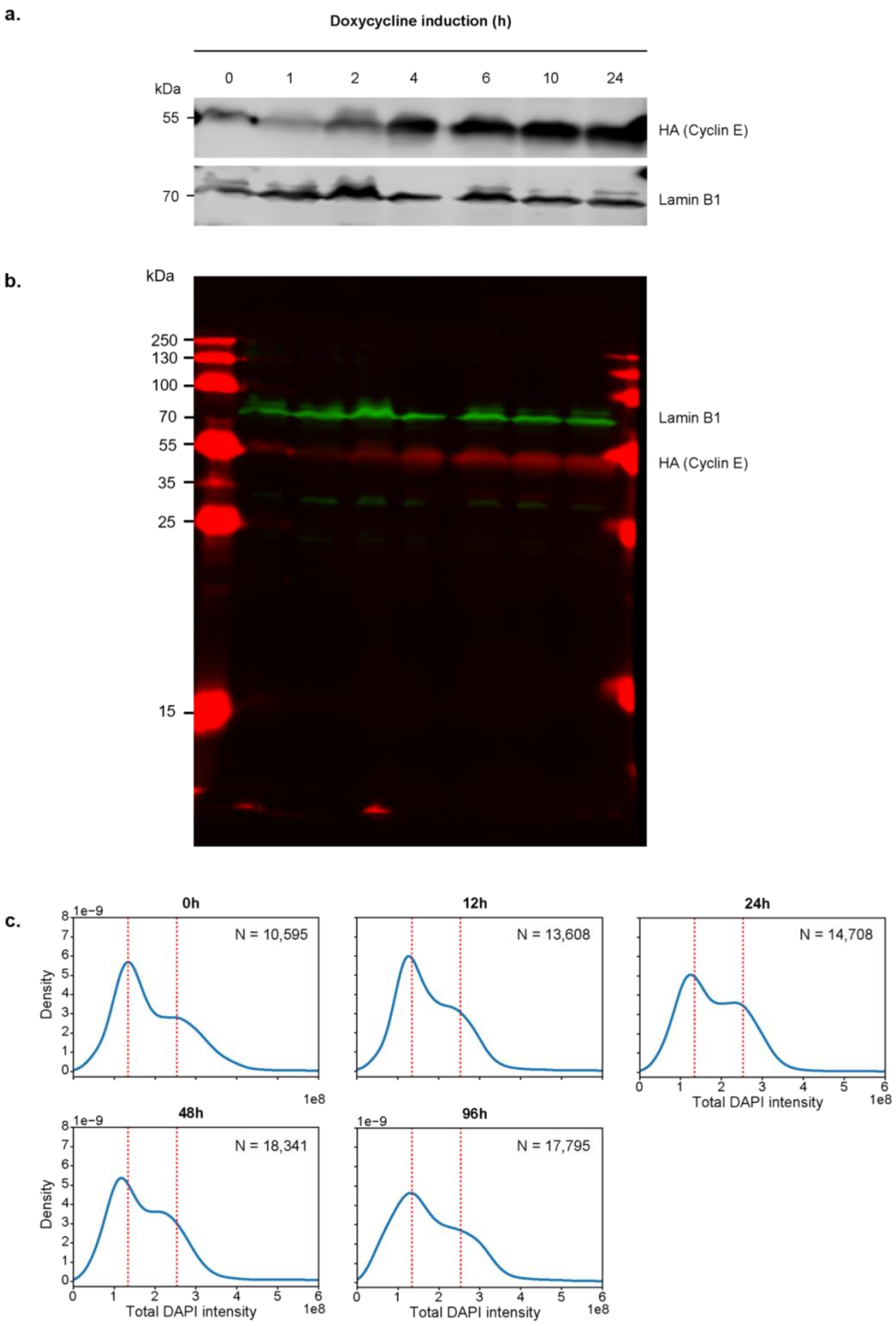

Supplementary Fig. 7. Doxycycline-induced expression of cyclin E1. a. Expression of cyclin E1-HA in U2OS TetON CycE cells was detected by immunoblots with anti HA-antibody. Lamin B1 was used as a loading control. **b.** Original, uncropped LICOR scan from a. **c.** KDE plots of the total DAPI intensity at each timepoint. The number of nuclei corresponding to each timepoint is shown. Vertical dotted lines are drawn in red at the approximate G1 and G2 positions (based on the '0h' timepoint) to facilitate comparison across timepoints.

a.

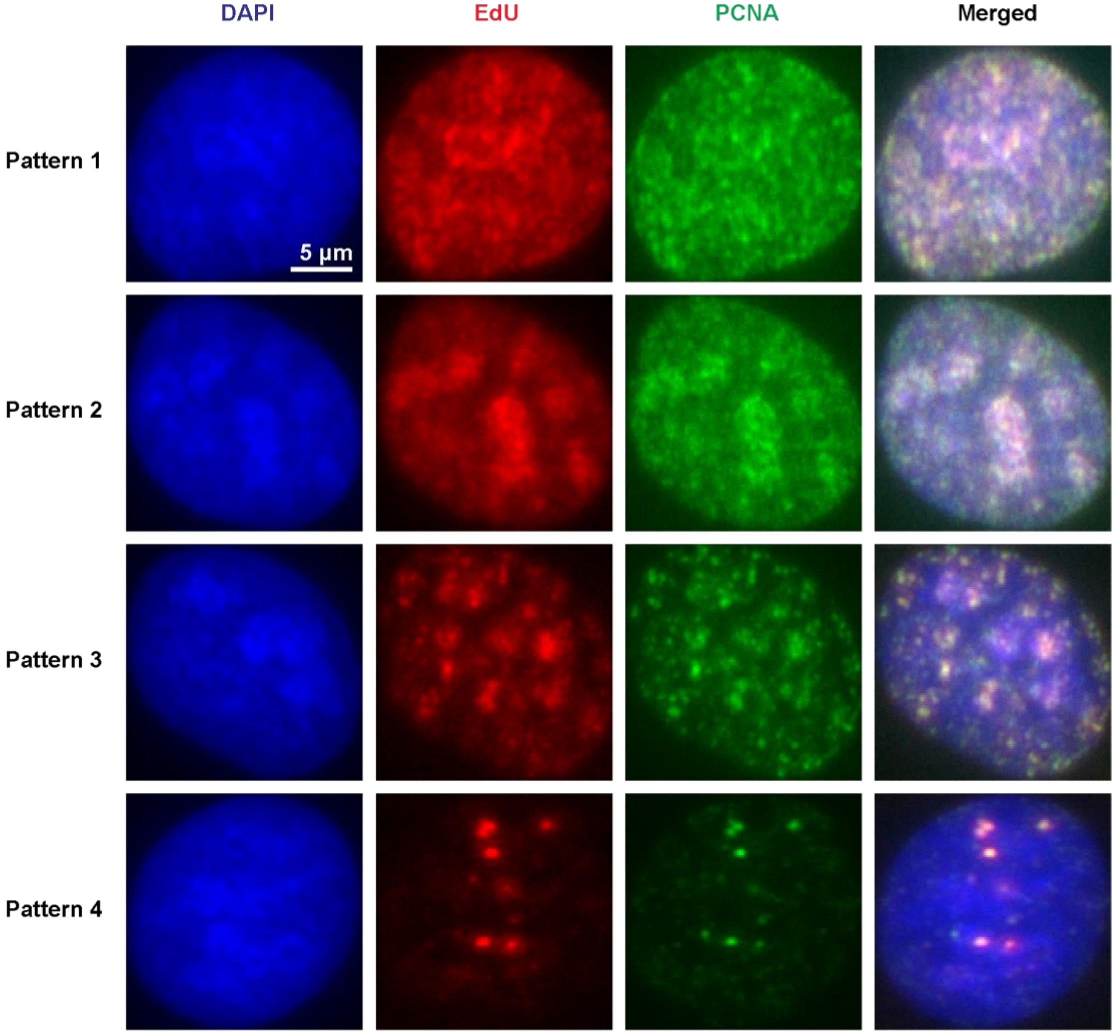

b.

Timepoint	N
0h	2,443
12h	5,912
24h	5,001
48h	7,106
96h	5,675

Supplementary Fig. 8. U2OS S-phase nuclei. a. Representative images of S-phase nuclei corresponding to four different observed patterns in U2OS cells (not treated with doxycycline) are shown. Images were taken with an Olympus ScanR High Content Screening Microscope, using a 40x objective. **b.** Table summarising the number of S-phase nuclei analysed per time point. In total, we identified 26,137 S-phase nuclei following segmentation and filtering steps.

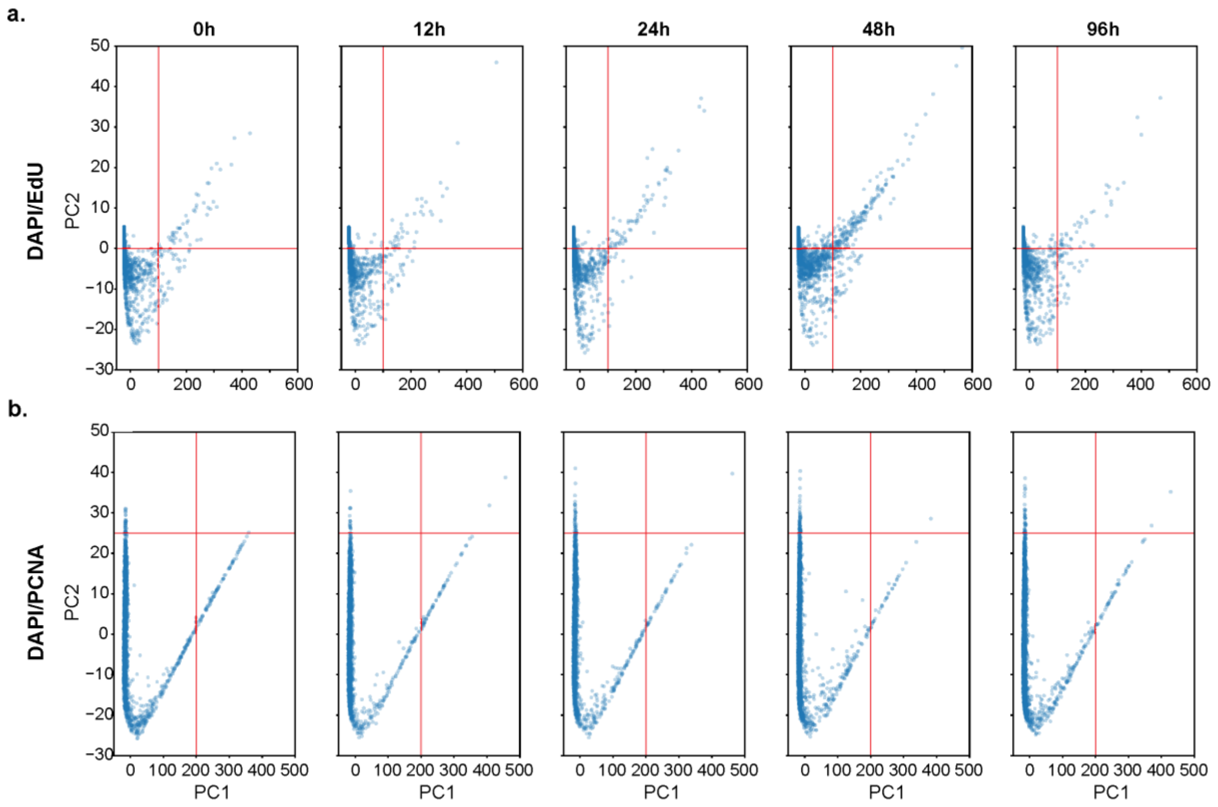

Supplementary Fig. 9. Deregulated origin firing in U2OS TetON CycE cells identified based on EdU and PCNA. Scatter plots of the projected image embeddings, corresponding to the KDE plots in **a.** Fig. 5a. and **b.** Fig. 5b. An equal number of nuclei per timepoint (N = 2,000) was randomly sampled to facilitate comparison.

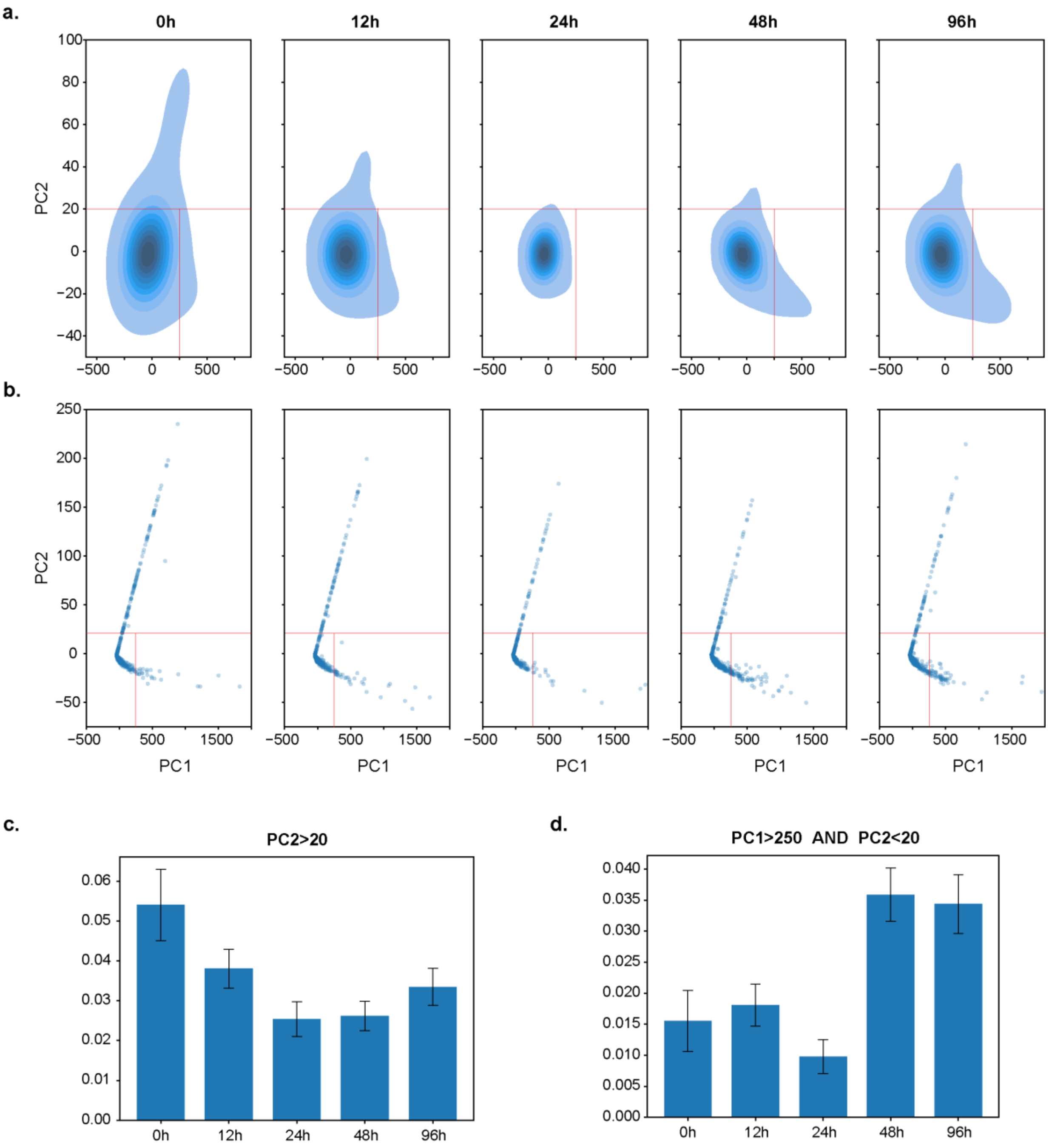

Supplementary Fig. 10. Deregulated origin firing in U2OS TetON CycE cells identified by integrating information from DAPI, EdU and PCNA channels. a. KDE plots and **b.** Scatter plots of the projected image embeddings obtained from the model trained on images including all three channels. For the scatterplots in **b.**, an equal number of nuclei per timepoint (N = 2,000) was randomly sampled to facilitate comparison. **c.** Bar plots showing the proportion of nuclei, per timepoint, with PC2 values greater than 20. P-values for differences in proportion following addition of doxycycline are as follows: 12h: $p = 1.0 \times 10^{-3}$; 24h: $p = 2.4 \times 10^{-10}$; 48h: $p = 3.6 \times 10^{-11}$; 96h: $p = 1.4 \times 10^{-5}$. **d.** Bar plots showing the proportion of nuclei, per timepoint, with both PC1 values greater than 250 and PC2 values less than 20. P-values for differences in proportion following addition of doxycycline are as follows for **d**: 12h: $p = 0.42$; 24h: $p = 0.03$; 48h: $p = 5.0 \times 10^{-7}$; 96h: $p = 3.2 \times 10^{-6}$. Differences in proportion between the '0h' timepoint

and other timepoints were assessed using two-proportion Z-tests. The error bars in **c.** and **d.** represent 95% confidence intervals.

a.

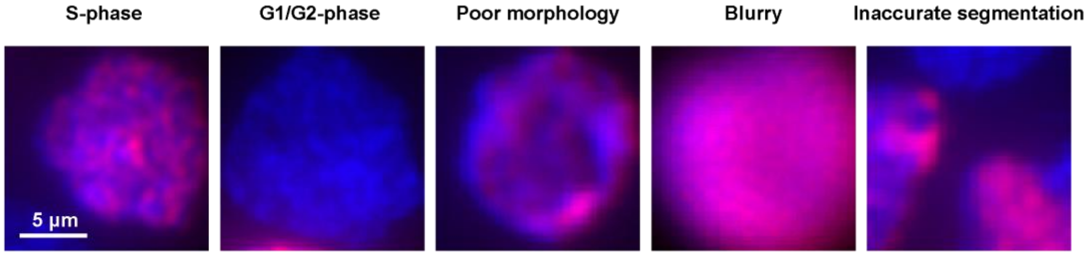

b.

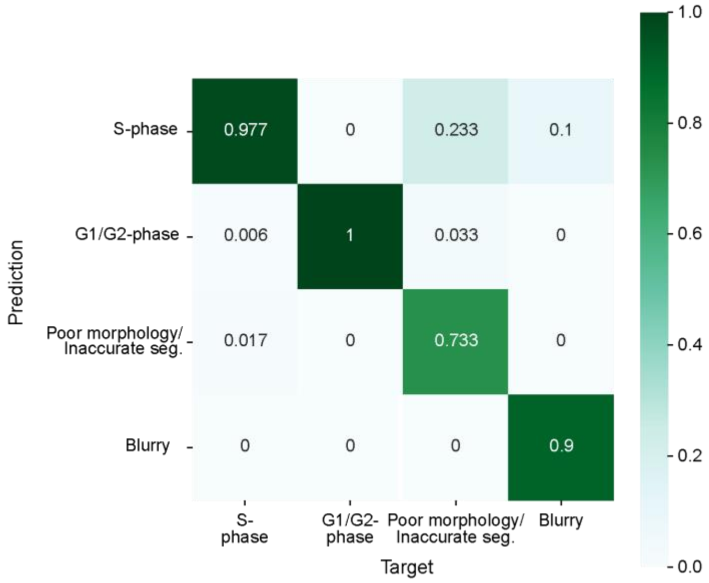

c.

	Genotype	N
–OHT	Rif1 $^{WT/WT}$	4,941
	Rif1 $^{TgWT/flox}$	5,487
	Rif1 $^{\Delta PP1/flox}$	9,004
	Rif1 $^{flox/flox}$	7,994
+OHT	Rif1 $^{WT/WT}$ (+OHT)	10,174
	Rif1 $^{TgWT/-}$	1,745
	Rif1 $^{\Delta PP1/-}$	3,500
	Rif1 $^{-/-}$	2,283

Supplementary Fig. 11. Use of a convolutional neural network ('overall classifier') to identify S-phase nuclei across the whole dataset. a. Representative images corresponding each of the five possible categories encountered during manual inspection of the 1,202 images retained following image segmentation and filtering. These 1,202 manually categorised images were used for training and testing a convolutional neural network ('overall classifier'), whose aim was to identify S-phase nuclei across the whole dataset comprising both wild-type and mutant cell lines. The blue and red channels correspond to DAPI and EdU, respectively. **b.** Confusion matrix showing the performance of a pre-trained ResNet-50 convolutional neural network in identifying S-phase nuclei. Images of nuclei demonstrating poor morphology and those corresponding to inaccurate segmentation were considered as a single class given the small number of images present in the latter category. **c.** Table showing the number of S-phase nuclei, per genotype, present in our dataset. In total, we identified 45,128 S-phase nuclei across the full dataset following segmentation and filtering steps.
