# Supplementary material for: Supervised and unsupervised deep learning-based approaches for studying DNA replication spatiotemporal dynamics
Source: Commun Biol. 2025 Feb 26;8:311. doi: 10.1038/s42003-025-07744-2 (PMC11865476; doi:10.1038/s42003-025-07744-2)
Supplement: Supplementary file 2 — Reporting Summary [file 42003_2025_7744_MOESM2_ESM.pdf]

nature portfolio

Corresponding author(s): Sara C. B. Buonomo

Last updated by author(s): Feb 7, 2025

Reporting Summary

Nature Portfolio wishes to improve the reproducibility of the work that we publish. This form provides structure for consistency and transparency in reporting. For further information on Nature Portfolio policies, see our Editorial Policies and the Editorial Policy Checklist.

Statistics

For all statistical analyses, confirm that the following items are present in the figure legend, table legend, main text, or Methods section.

n/a	Confirmed	
☐	☒	The exact sample size (n) for each experimental group/condition, given as a discrete number and unit of measurement
☐	☒	A statement on whether measurements were taken from distinct samples or whether the same sample was measured repeatedly
☐	☒	The statistical test(s) used AND whether they are one- or two-sided<br>*Only common tests should be described solely by name; describe more complex techniques in the Methods section.*
☒	☐	A description of all covariates tested
☐	☒	A description of any assumptions or corrections, such as tests of normality and adjustment for multiple comparisons
☐	☒	A full description of the statistical parameters including central tendency (e.g. means) or other basic estimates (e.g. regression coefficient) AND variation (e.g. standard deviation) or associated estimates of uncertainty (e.g. confidence intervals)
☐	☒	For null hypothesis testing, the test statistic (e.g. F, t, r) with confidence intervals, effect sizes, degrees of freedom and P value noted<br>*Give P values as exact values whenever suitable.*
☒	☐	For Bayesian analysis, information on the choice of priors and Markov chain Monte Carlo settings
☒	☐	For hierarchical and complex designs, identification of the appropriate level for tests and full reporting of outcomes
☒	☐	Estimates of effect sizes (e.g. Cohen's d, Pearson's r), indicating how they were calculated

Our web collection on statistics for biologists contains articles on many of the points above.

Software and code

Policy information about availability of computer code

Data collection	Images of cells were acquired at 40x magnification using the Olympus ScanR High Content Screening Microscope.
Data analysis	Scripts were written using Python version 3.7.12, with neural networks developed using PyTorch version 1.13.1 (CUDA version 11.7). Scientific analysis was carried out using NumPy version 1.21.6, pandas version 1.3.5, SciPy version 1.7.3 and scikit-learn version 1.0.2. Plots were made using Matplotlib 3.5.3 and Seaborn 0.12.1. Image analysis was performed using scikit-image version 0.19.3.

For manuscripts utilizing custom algorithms or software that are central to the research but not yet described in published literature, software must be made available to editors and reviewers. We strongly encourage code deposition in a community repository (e.g. GitHub). See the Nature Portfolio guidelines for submitting code & software for further information.

Data

Policy information about availability of data

All manuscripts must include a data availability statement. This statement should provide the following information, where applicable:

- Accession codes, unique identifiers, or web links for publicly available datasets
- A description of any restrictions on data availability
- For clinical datasets or third party data, please ensure that the statement adheres to our policy

The image datasets associated with this paper have been deposited at Edinburgh Datashare, which is a digital repository of research data produced at the University of Edinburgh. They are publicly available at the following: https://doi.org/10.7488/ds/7754

Research involving human participants, their data, or biological material

Policy information about studies with human participants or human data. See also policy information about sex, gender (identity/presentation), and sexual orientation and race, ethnicity and racism.

Reporting on sex and gender	This study did not involve human participants or human data.
Reporting on race, ethnicity, or other socially relevant groupings	This study did not involve human participants or human data.
Population characteristics	This study did not involve human participants or human data.
Recruitment	This study did not involve human participants or human data.
Ethics oversight	This study did not involve human participants or human data.

Note that full information on the approval of the study protocol must also be provided in the manuscript.

Field-specific reporting

Please select the one below that is the best fit for your research. If you are not sure, read the appropriate sections before making your selection.

☒ Life sciences ☐ Behavioural & social sciences ☐ Ecological, evolutionary & environmental sciences

For a reference copy of the document with all sections, see nature.com/documents/nr-reporting-summary-flat.pdf

Life sciences study design

All studies must disclose on these points even when the disclosure is negative.

Sample size	No formal sample size calculation was performed for this study. However, similar analyses involving image annotation are generally based on smaller datasets comprising manually acquired images. Here, we leveraged automated microscopy to generate a considerably larger image dataset, given the need to train machine learning models.
Data exclusions	No cell line/sample was excluded from our analysis. Filtering and segmentation steps (described in the 'Methods' section) were performed to identify nuclei deemed suitable for manual categorisation. The same filtering criteria were applied across all cell lines.
Replication	Two clones per genotype were included as biological replicates. Comparable results were observed for both clones (See Supplementary Fig. 4b and Supplementary Fig. 6b)
Randomization	For supervised learning, the manually categorised dataset was randomly split with stratified sampling into training, validation and test set according to a 60:20:20 ratio.
Blinding	Blinding was not relevant to our study given that image analysis was performed by machine learning models, thus avoiding human subjectivity.

Reporting for specific materials, systems and methods

We require information from authors about some types of materials, experimental systems and methods used in many studies. Here, indicate whether each material, system or method listed is relevant to your study. If you are not sure if a list item applies to your research, read the appropriate section before selecting a response.

Materials & experimental systems

n/a	Involved in the study	
☐	☒	Antibodies
☐	☒	Eukaryotic cell lines
☒	☐	Palaeontology and archaeology
☒	☐	Animals and other organisms
☒	☐	Clinical data
☒	☐	Dual use research of concern
☒	☐	Plants

Methods

n/a	Involved in the study	
☒	☐	ChIP-seq
☐	☒	Flow cytometry
☒	☐	MRI-based neuroimaging

Antibodies

Antibodies used	Anti-PCNA (Abcam, ab29 [PC10]); Anti-HA (Covance monoclonal HA.11 clone 16B12 #MMS-101R); Anti-lamin B1 (Abcam #ab16048)
Validation	Anti-PCNA antibody has been used in the publication doi:10.1038/ncb2918 from one of the authors of this paper (C.A.). Covance monoclonal HA.11 clone 16B12 #MMS-101R has been used by our lab previously and is validated in the western blot by the presence of a negative (untagged) protein. Abcam #ab16048 anti-Lamin B1 has been used previously by our group. It is KO validated (Abcam webpage)

Eukaryotic cell lines

Policy information about cell lines and Sex and Gender in Research

Cell line source(s)	The cell lines used in this study have been previously described in the following publications:<br>- Gnan, S. et al. Nuclear organisation and replication timing are coupled through RIF1-PP1 interaction. Nat Commun 12, 2910, doi:10.1038/s41467-021-22899-2 (2021).<br>- Foti, R. et al. Nuclear Architecture Organized by Rif1 Underpins the Replication-Timing Program. Mol Cell 61, 260-273, doi:10.1016/j.molcel.2015.12.001 (2016).<br>- Buonomo, S. B., Wu, Y., Ferguson, D. & de Lange, T. Mammalian Rif1 contributes to replication stress survival and homology-directed repair. J Cell Biol 187, 385-398, doi:10.1083/jcb.200902039 (2009).<br>- Zeng, J., Hills, S. A., Ozono, E. & Diffley, J. F. X. Cyclin E-induced replicative stress drives p53-dependent whole-genome duplication. Cell 186, 528-542 e514, doi:10.1016/j.cell.2022.12.036 (2023).
Authentication	Cell line authentication is also described in the publications listed above.
Mycoplasma contamination	The cell lines in the lab are periodically randomly tested for mycoplasma contamination.
Commonly misidentified lines<br>(See ICLAC register)	N/A

Plants

Seed stocks	This study did not involve plants.
Novel plant genotypes	This study did not involve plants.
Authentication	This study did not involve plants.

Flow Cytometry

Plots

Confirm that:

☒ The axis labels state the marker and fluorochrome used (e.g. CD4-FITC).

☒ The axis scales are clearly visible. Include numbers along axes only for bottom left plot of group (a 'group' is an analysis of identical markers).

☒ All plots are contour plots with outliers or pseudocolor plots.

☒ A numerical value for number of cells or percentage (with statistics) is provided.

Methodology

Sample preparation	samples were pulsed for 30 minutes with EdU, fixed with 2% PFA for 10 minutes and stained over night in saponin buffer with DAPI.
Instrument	LSRFortessa(SORP-BD)
Software	FACSDiva 8.0
Cell population abundance	Intact cells are typically 85-95% of the total population, with singlets representing 85-90% of the intact population.

Gating strategy	Intact cells were selected based on the FSCA-A and SSC-A distributions, while singlets were selected out of the intact cells by gating for DAPiI-A and W.

☒ Tick this box to confirm that a figure exemplifying the gating strategy is provided in the Supplementary Information.
