# Supplementary material for: Supervised and unsupervised deep learning-based approaches for studying DNA replication spatiotemporal dynamics
Source: Commun Biol. 2025 Feb 26;8:311. doi: 10.1038/s42003-025-07744-2 (PMC11865476; doi:10.1038/s42003-025-07744-2)
Supplement: Supplementary file 3 — Transparent Peer Review file [file 42003_2025_7744_MOESM3_ESM.pdf]

nature portfolio

Peer Review File

Supervised and unsupervised deep learning-based approaches for studying DNA replication spatiotemporal dynamics

Corresponding Author: Dr Sara Buonomo

This manuscript has been previously reviewed at another journal that is not operating a transparent peer review scheme. The manuscript was considered suitable for publication without further review at Communications Biology.

Open Access This Peer Review File is licensed under a Creative Commons Attribution 4.0 International License, which permits use, sharing, adaptation, distribution and reproduction in any medium or format, as long as you give appropriate credit to the original author(s) and the source, provide a link to the Creative Commons license, and indicate if changes were made.
In cases where reviewers are anonymous, credit should be given to 'Anonymous Referee' and the source.
The images or other third party material in this Peer Review File are included in the article's Creative Commons license, unless indicated otherwise in a credit line to the material. If material is not included in the article's Creative Commons license and your intended use is not permitted by statutory regulation or exceeds the permitted use, you will need to obtain permission directly from the copyright holder.
To view a copy of this license, visit https://creativecommons.org/licenses/by/4.0/
